# Exploring the Cutting Edge of Vision Science: New Developments in Diagnostics and Treatment of Ocular Surface in Dry Eye Disease

**DOI:** 10.3390/life13071584

**Published:** 2023-07-19

**Authors:** José-María Sánchez-González, Carlos Rocha-de-Lossada, Alejandro Cerviño

**Affiliations:** 1 Department of Physics of Condensed Matter, Optics Area, University of Seville, 41012 Seville, Spain; 2 Qvision, Ophthalmology Department, VITHAS Almeria Hospital, 04120 Almeria, Spain; 3 Ophthalmology Department, VITHAS Malaga, 29016 Malaga, Spain; 4 Regional University Hospital of Malaga, Hospital Civil Square, 29009 Malaga, Spain; 5 Surgery Department, Ophthalmology Area, University of Seville, Doctor Fedriani, 41009 Seville, Spain; 6 Department of Optics & Optometry & Vision Sciences, University of Valencia, 46010 Valencia, Spain

The ocular surface refers to the outermost layer of the eye, which includes the cornea, conjunctiva and eyelids [1]. It is a complex and delicate system that is responsible for maintaining the health and function of the eye [2]. One common problem that can affect the ocular surface is dry eye syndrome, which is a multifactorial condition that occurs when the eye does not produce enough tears or the tears produced do not have the correct balance of water, mucus and oil, where inflammation, hyperosmolarity and neurosensorial abnormalities could coexist [3]. Dry eye syndrome can be caused by a variety of factors, including aging, certain medications and environmental conditions, such as prolonged use of the screen or living in a dry or dusty environment [3], or be related to autoimmune syndromes, where Sjögren syndrome stands out [4]. Symptoms and signs of dry eye syndrome include burning, itching or redness of the eye, as well as a sensation of dryness, grittiness or a foreign body sensation, among others. In severe cases, dry eye syndrome can cause vision problems, corneal damage and even blindness [5,6]. Effective treatment of dry eye syndrome requires an accurate diagnosis and a personalized treatment plan. Currently, the most common subjective method of diagnosing dry eye syndrome is through the use of questionnaires and subjective tests, such as the Ocular Surface Disease Index (OSDI) [7] or the Dry Eye Questionnaire (DEQ) [7]. Although these tests can be useful in identifying the presence of dry eye syndrome, they are based on self-reported symptoms and may not accurately reflect the true severity of the condition [7]. Moreover, traditional objective tests include the Oxford grading system with fluorescein stain or lissamine green stain, the tear break-up time test (TBUT) or the Schirmer test (I and II) [1,2,3,4,5,6,7,8,9,10]. Other adjuvant options are the measurement of the tear meniscus [11], the evaluation of the metalloproteinases [12] or the tear osmolarity [13].

A new measurement device, called the Ocular Surface Analyzer (OSA) [14], has recently been developed to help improve the diagnosis and management of dry eye syndrome. The OSA is a non-invasive, objective tool that uses interferometry to measure the thickness and surface profile of the tear film, similar to a Keratograph [15]. It can also measure blink rate and eyelid position, as well as TBUT. One of the key advantages of the OSA is its ability to provide detailed, quantitative data about the ocular surface [14]. This allows for a more accurate diagnosis of dry eye syndrome and allows for a more personalized treatment plan to be developed [16]. The OSA can also be used to monitor the effectiveness of treatment and track changes in the ocular surface over time [17]. There is growing evidence to suggest that OSA is a valuable tool in the diagnosis and management of dry eye syndrome [18]. A recent study found that OSA was able to accurately detect changes in the ocular surface in patients with dry eye syndrome and that it was able to distinguish between different severities of the condition [19]. Another study found that OSA was able to accurately predict the presence of dry eye syndrome in patients who had previously been diagnosed with the condition [20].

In general, OSA appears to be a promising new tool for the diagnosis and treatment of dry eye syndrome. Its non-invasive, objective measurements provide detailed, quantitative data about the ocular surface, which can help to improve the accuracy of diagnosis and the effectiveness of treatment. As research on OSA and its potential uses continues to be conducted, it is likely to become an increasingly valuable tool in the treatment of dry eye syndrome and other conditions that affect the ocular surface.

Second, dry eye disease is a common condition that occurs when the eye does not produce enough tears or the tears evaporate too quickly [21]. This can lead to a variety of symptoms, including dryness, irritation, redness and a feeling of discomfort or a foreign body sensation [7]. In severe cases, it can even cause vision problems [5]. The stability of the tear film is an important factor in the health and comfort of the eye [22,23,24]. The tear film is a thin layer of moisture that coats the surface of the eye and helps to keep it lubricated and protected [25]. It is made up of three layers: the outer layer, which is composed of oil produced by the meibomian glands [26,27]; the middle layer, which is made up of water produced by the lacrimal glands; and the inner layer, which is composed of mucus produced by the conjunctiva [10].

The tear film plays a critical role in maintaining the health of the eye by providing a protective barrier against dust, dirt and other irritants. It also helps keep the eye surface moist and comfortable, which is essential for good vision [6]. There are several factors that can affect tear film stability, including age, hormonal changes [16,28,29], medications [30,31] and certain medical conditions. Dry eye disease is one of the most common causes of tear film instability and is more common in women than in men, especially after menopause [16,28,29]. To treat dry eye disease, doctors may recommend the use of eye drops or ointments to supplement the natural tear film [6,9]. There are several types of eyedrops available, including artificial tears, which are designed to mimic the natural tear film; lubricating drops, which help to moisturize the eye; and anti-inflammatory drops, which reduce inflammation and redness [28,29,30,31,32,33].

In recent years, there has been a significant amount of research into the development of new eyedrop formulations that are more effective in treating dry eye disease. One promising area of research is the use of lipid-based eyedrops, which are designed to mimic the natural tear film more closely [33]. These drops are composed of a mixture of oils and water, and they are able to stay on the eye longer than traditional artificial tears. Another area of research is the use of nanotechnology to create eyedrops with smaller particle sizes that can be absorbed more easily by the eye [32]. These drops have the potential to provide more sustained relief from dry eye symptoms and may be more effective in improving tear film stability [22]. In general, dry eye disease is a common and often debilitating condition that can cause a variety of symptoms, including dryness, irritation and vision problems. Maintaining the stability of the tear film is an important factor in maintaining eye health and comfort, and new eyedrop formulations are being developed to improve the treatment of dry eye disease [34,35,36,37,38,39,40]. In more severe cases, other options, such as the use of corticosteroids, autologous or allogenic serum [41], immunomodulators or secretagogues and even surgical approaches [42], are necessary [6].

On the last point of discussion for this Editorial, the ocular surface microbiota, or the collection of microorganisms living on the surface of the eye, has long been recognized as an important factor in the health and function of the eye [43]. However, the specific role of the ocular surface microbiota in the development and management of eye diseases is still not fully understood [44]. Recently, a multicenter study proposed the concept of eye community state type (ECST) as a way to categorize and understand the different profiles of bacterial communities that can exist in the healthy eye [43]. The study found that nine different ECSTs could be identified within the healthy bacterial population. This is an exciting finding, as it suggests that there may be multiple “healthy” states of the ocular surface microbiota and that different individuals may have different ECSTs. It also opens up the possibility of developing personalized approaches to eye care based on an individual’s ECST.

However, more research is needed to fully understand the clinical implications of ECST and how it may be related to the development and management of eye diseases [45,46,47,48]. For example, it is not yet known whether certain ECSTs are more or less prone to developing eye infections or other problems. Overall, the concept of ECST is an interesting new avenue for research on the ocular surface microbiota and its role in eye health [49,50,51]. Further studies are needed to fully understand the clinical importance of ECST and how it can be used to improve the diagnosis and treatment of eye diseases. The field of vision science is constantly evolving, and there have been many exciting developments in the diagnosis and treatment of the ocular surface in dry eye disease. New technologies and approaches are being developed that have the potential to greatly improve the lives of those suffering from this common and often debilitating condition.

An important area of research is the development of new eyedrop formulations that are more effective at treating dry eye disease. Lipid-based eyedrops, which mimic the natural tear film, and nanotechnology-based drops with smaller particle sizes, which can be absorbed more easily by the eye, are both promising approaches that have the potential to provide more sustained relief from dry eye symptoms and improve tear film stability. In addition, advances in diagnostic techniques, such as the use of non-invasive imaging techniques, are helping to improve the accuracy and reliability of dry eye diagnoses. This is important because it allows physicians to more effectively tailor treatment plans to the specific needs of each patient.

In general, the cutting edge of vision science provides new and innovative ways to diagnose and treat ocular surface conditions, including dry eye disease. These developments have the potential to greatly improve the lives of those affected by this condition and to help them maintain the health and comfort of their eyes.

## References

[1] Nelson J.D., Craig J.P., Akpek E.K., Azar D.T., Belmonte C., Bron A.J., Clayton J.A., Dogru M., Dua H.S., Foulks G.N. (2017). TFOS DEWS II Introduction. Ocul. Surf..

[2] Craig J.P., Nichols K.K., Akpek E.K., Caffery B., Dua H.S., Joo C.-K., Liu Z., Nelson J.D., Nichols J.J., Tsubota K. (2017). TFOS DEWS II Definition and Classification Report. Ocul. Surf..

[3] Stapleton F., Alves M., Bunya V.Y., Jalbert I., Lekhanont K., Malet F., Na K.-S., Schaumberg D., Uchino M., Vehof J. (2017). TFOS DEWS II Epidemiology Report. Ocul. Surf..

[4] Barrientos R.T., Godín F., Rocha-De-Lossada C., Soifer M., Sánchez-González J.-M., Moreno-Toral E., González A.-L., Zein M., Larco P., Mercado C. (2022). Ophthalmological Approach for the Diagnosis of Dry Eye Disease in Patients with Sjögren’s Syndrome. Life.

[5] Bron A.J., de Paiva C.S., Chauhan S.K., Bonini S., Gabison E.E., Jain S., Knop E., Markoulli M., Ogawa Y., Perez V. (2017). TFOS DEWS II pathophysiology report. Ocul. Surf..

[6] Jones L., Downie L.E., Korb D., Benitez-Del-Castillo J.M., Dana R., Deng S.X., Dong P.N., Geerling G., Hida R.Y., Liu Y. (2017). TFOS DEWS II Management and Therapy Report. Ocul. Surf..

[7] Wolffsohn J.S., Arita R., Chalmers R., Djalilian A., Dogru M., Dumbleton K., Gupta P.K., Karpecki P., Lazreg S., Pult H. (2017). TFOS DEWS II Diagnostic Methodology report. Ocul. Surf..

[8] Belmonte C., Nichols J.J., Cox S.M., Brock J.A., Begley C.G., Bereiter D.A., Dartt D.A., Galor A., Hamrah P., Ivanusic J.J. (2017). TFOS DEWS II pain and sensation report. Ocul. Surf..

[9] Craig J.P., Nelson J.D., Azar D.T., Belmonte C., Bron A.J., Chauhan S.K., de Paiva C.S., Gomes J.A.P., Hammitt K.M., Jones L. (2017). TFOS DEWS II Report Executive Summary. Ocul. Surf..

[10] Willcox M.D.P., Argüeso P., Georgiev G.A., Holopainen J.M., Laurie G.W., Millar T.J., Papas E.B., Rolland J.P., Schmidt T.A., Stahl U. (2017). TFOS DEWS II Tear Film Report. Ocul. Surf..

[11] Rocha-de-Lossada C., Sánchez-González J.M., Zamorano-Martín F., Rachwani-Anil R., Torras-Sanvicens J., Peraza-Nieves J. (2021). Influence of Sodium Hyaluronate Concentration in Tear Meniscus Height: 10-min Dynamic Profile After Single Instillation. Eye Contact Lens.

[12] Huh J., Choi S.Y., Eom Y., Kim H.M., Song J.S. (2020). Changes in the Matrix Metalloproteinase 9 Point-of-Care Test Positivity According to MMP-9 Concentration and Loading Volume. Cornea.

[13] Lemp M.A., Bron A.J., Baudouin C., Bentez Del Castillo J.M., Geffen D., Tauber J., Foulks G.N., Pepose J.S., Sullivan B.D. (2011). Tear osmolarity in the diagnosis and management of dry eye disease. Am. J. Ophthalmol..

[14] Sánchez-González M.C., Capote-Puente R., García-Romera M.-C., De-Hita-Cantalejo C., Bautista-Llamas M.-J., Silva-Viguera C., Sánchez-González J.-M. (2022). Dry eye disease and tear film assessment through a novel non-invasive ocular surface analyzer: The OSA protocol. Front. Med..

[15] García-Marqués J.V., Martínez-Albert N., Talens-Estarelles C., García-Lázaro S., Cerviño A. (2021). Repeatability of Non-invasive Keratograph Break-Up Time measurements obtained using Oculus Keratograph 5M. Int. Ophthalmol..

[16] García-Marqués J.V., Talens-Estarelles C., García-Lázaro S., Wolffsohn J.S., Cerviño A. (2022). Systemic, environmental and lifestyle risk factors for dry eye disease in a mediterranean caucasian population. Contact Lens Anterior Eye.

[17] Singh S., Srivastav S., Modiwala Z., Ali M.H., Basu S. (2022). Repeatability, reproducibility and agreement between three different diagnostic imaging platforms for tear film evaluation of normal and dry eye disease. Eye.

[18] Lee J.M., Jeon Y.J., Kim K.Y., Hwang K.-Y., Kwon Y.-A., Koh K. (2021). Ocular surface analysis: A comparison between the LipiView^®^ II and IDRA^®^. Eur. J. Ophthalmol..

[19] Rinert J., Branger G., Bachmann L.M., Pfaeffli O., Iselin K., Kaufmann C., Thiel M.A., Baenninger P.B. (2022). Accuracy of a New Noninvasive Automatic Ocular Surface Analyzer for the Diagnosis of Dry Eye Disease-Two-Gate Design Using Healthy Controls. Cornea.

[20] Yadav S., Gupta N., Makwana T., Vanathi M., Tandon R. (2022). Noninvasive ocular surface analyzer as an adjunct in diagnosis and estimating prevalence of meibomian gland dysfunction: Hospital-based comparative study. Indian J. Ophthalmol..

[21] Montés-Micó R., Cerviño A., Ferrer-Blasco T., García-Lázaro S., Madrid-Costa D. (2010). The tear film and the optical quality of the eye. Ocul. Surf..

[22] Montés-Micó R., Cerviño A., Ferrer-Blasco T., García-Lázaro S., Ortí-Navarro S. (2010). Optical quality after instillation of eyedrops in dry-eye syndrome. J. Cataract Refract. Surg..

[23] García-Marqués J.V., Macedo-De-Araújo R.J., McAlinden C., Faria-Ribeiro M., Cerviño A., González-Méijome J.M. (2022). Short-term tear film stability, optical quality and visual performance in two dual-focus contact lenses for myopia control with different optical designs. Ophthalmic Physiol. Opt. J. Br. Coll. Ophthalmic Opt..

[24] Capote-Puente R., Eftimov P., Bautista-Llamas M.-J., Yokoi N., Sánchez-González J.-M., Georgiev G. (2022). Short-term tear film stability, optical quality and visual performance in two dual-focus contact lenses for myopia control with different optical designs. Ophthalmic Physiol. Opt. J. Br. Coll. Ophthalmic Opt..

[25] Vidal-Rohr M., Wolffsohn J.S., Davies L.N., Cerviño A. (2018). Effect of contact lens surface properties on comfort, tear stability and ocular physiology. Cont. Lens Anterior Eye.

[26] García-Marqués J.V., García-Lázaro S., Martínez-Albert N., Cerviño A. (2021). Meibomian glands visibility assessment through a new quantitative method. Graefe’s Arch. Clin. Exp. Ophthalmol..

[27] García-Marqués J.V., Talens-Estarelles C., García-Lázaro S., Cerviño A. (2022). Validation of a new objective method to assess lipid layer thickness without the need of an interferometer. Graefe’s Arch. Clin. Exp. Ophthalmol..

[28] De-Hita-Cantalejo C., Sánchez-González M.C., Silva-Viguera C., García-Romera M.C., Feria-Mantero R., Sánchez-González J.-M. (2022). Efficacy of hyaluronic acid 0.3%, cyanocobalamin, electrolytes, and P-Plus in menopause patients with moderate dry eye disease. Graefe’s Arch. Clin. Exp. Ophthalmol..

[29] Serrano-Morales J.-M., De-Hita-Cantalejo C., Sánchez-González M.C., Bautista-Llamas M.-J., Sánchez-González J.-M. (2022). Efficacy of 0.1% crosslinked hyaluronic acid, coenzyme Q10 and vitamin E in the management of dry eye disease in menopause patients receiving antidepressants. Eur. J. Ophthalmol..

[30] Sánchez-González M.C., De-Hita-Cantalejo C., Martínez-Lara C., Sánchez-González J.-M. (2022). Oral isotretinoin for acne vulgaris side effects on the ocular surface: Hyaluronic acid and galacto-xyloglucan as treatment for dry eye disease signs and symptoms. Front. Med..

[31] Sánchez-González J.-M., De-Hita-Cantalejo C., Sánchez-González M.C. (2022). Hyaluronic Acid and Galacto-Xyloglucan Eyedrop Efficacy in Young-Adult Oral Contraceptive Users of Childbearing Age. J. Clin. Med..

[32] Sánchez-González J.-M., De-Hita-Cantalejo C., Sánchez-González M.C. (2020). Crosslinked hyaluronic acid with liposomes and crocin for management symptoms of dry eye disease caused by moderate meibomian gland dysfunction. Int. J. Ophthalmol..

[33] Sánchez-González J.-M., De-Hita-Cantalejo C., Martínez-Lara C., Sánchez-González M.C. (2022). Lipid, Aqueous and Mucin Tear Film Layer Stability and Permanence within 0.15% Liposome Crosslinked Hyaluronic Acid versus 0.15% Non-Crosslinked Hyaluronic Acid Measured with a Novel Non-Invasive Ocular Surface Analyzer. J. Clin. Med..

[34] Talens-Estarelles C., García-Marqués J.V., Cerviño A., García-Lázaro S. (2022). Determining the Best Management Strategy for Preventing Short-Term Effects of Digital Display Use on Dry Eyes. Eye Contact Lens.

[35] Szczesna-Iskander D.H., Muzyka-Wozniak M., Llorens Quintana C. (2022). The efficacy of ocular surface assessment approaches in evaluating dry eye treatment with artificial tears. Sci. Rep..

[36] Chen N., Zhang J.-S., Zhang T.-X., Fan B.-L., Ning Y. (2022). The effect of sodium hyaluronate on tear film stability in patients with dry eye syndrome after cataract surgery. Graefe’s Arch. Clin. Exp. Ophthalmol..

[37] Srinivasan S., Williams R. (2022). Propylene Glycol and Hydroxypropyl Guar Nanoemulsion—Safe and Effective Lubricant Eye Drops in the Management of Dry Eye Disease. Clin. Ophthalmol..

[38] Roszkowska A.M., Spinella R., Oliverio G.W., Postorino E.I., Signorino G.A., Rusciano D., Aragona P. (2022). Effects of the Topical Use of the Natural Antioxidant Alpha-Lipoic Acid on the Ocular Surface of Diabetic Patients with Dry Eye Symptoms. Front. Biosci. Landmark Ed..

[39] Murtaza F., Toameh D., Chiu H.H., Tam E.S., Somani S. (2022). Autologous Platelet-Rich Plasma Drops for Evaporative Dry Eye Disease from Meibomian Gland Dysfunction: A Pilot Study. Clin. Ophthalmol..

[40] Montani G., Landini L., Martino M. (2022). Short- and Long-Term Effects of a Multi-Component, Artificial Tear on Preocular Tear Film Stability, Tear Evaporation and Tear Film Optical Dynamic: A Prospective Randomized Double-Phase Study. Curr. Eye Res..

[41] Rodríguez Calvo-de-Mora M., Domínguez-Ruiz C., Barrero-Sojo F., Rodríguez-Moreno G., Antúnez Rodríguez C., Ponce Verdugo L., Hernández Lamas M.d.C., Hernández-Guijarro L., Villalvilla Castillo J., Fernández-Baca Casares I. (2022). Autologous versus allogeneic versus umbilical cord sera for the treatment of severe dry eye disease: A double-blind randomized clinical trial. Acta Ophthalmol..

[42] Messmer E.M., Ahmad S., Benitez Del Castillo J.M., Mrukwa-Kominek E., Rolando M., Vitovska O., Baudouin C., A Panel of European Dry Eye Disease Experts (2022). Management of inflammation in dry eye disease: Recommendations from a European panel of experts. Eur. J. Ophthalmol..

[43] Borroni D., Paytuví-Gallart A., Sanseverino W., Gómez-Huertas C., Bonci P., Romano V., Giannaccare G., Rechichi M., Meduri A., Oliverio G.W. (2022). Exploring the Healthy Eye Microbiota Niche in a Multicenter Study. Int. J. Mol. Sci..

[44] Gallon P., Parekh M., Ferrari S., Fasolo A., Ponzin D., Borroni D. (2019). Metagenomics in ophthalmology: Hypothesis or real prospective?. Biotechnol. Rep..

[45] Borroni D., Romano V., Kaye S.B., Somerville T., Napoli L., Fasolo A., Gallon P., Ponzin D., Esposito A., Ferrari S. (2019). Metagenomics in ophthalmology: Current findings and future prospectives. BMJ Open Ophthalmol..

[46] Hernández-Zulueta J., Navarro-Partida J., Sánchez-Aguilar O.E., Cruz-Pavlovich H.D.S., Castro-Castañeda C.R., la Rosa A.G.-D. (2022). An insight on the eye bacterial microbiota and its role on dry eye disease. APMIS.

[47] Tong L., Constancias F., Hou A., Chua S.L., Drautz-Moses D.I., Schuster S.C., Yang L., Williams R.B.H., Kjelleberg S. (2022). Shotgun metagenomic sequencing analysis of ocular surface microbiome in Singapore residents with mild dry eye. Front. Med..

[48] Watane A., Raolji S., Cavuoto K., Galor A. (2022). Microbiome and immune-mediated dry eye: A review. BMJ Open Ophthalmol..

[49] An Q., Zou H. (2022). Ocular surface microbiota dysbiosis contributes to the high prevalence of dry eye disease in diabetic patients. Crit. Rev. Microbiol..

[50] Jing D., Jiang X., Ren X., Su J., Huang C., Yang J., Hao R., Li X. (2022). Metagenomic nanopore sequencing of ocular microbiome in patients with meibomian gland dysfunction. Front. Med..

[51] Chen Z., Jia Y., Xiao Y., Lin Q., Qian Y., Xiang Z., Cui L., Qin X., Chen S., Yang C. (2022). Microbiological Characteristics of Ocular Surface Associated with Dry Eye in Children and Adolescents with Diabetes Mellitus. Investig. Ophthalmol. Vis. Sci..

